# Chronic rhinosinusitis and posterior cingulate hypoperfusion on SPECT in dementia diagnosis

**DOI:** 10.3389/fneur.2026.1777862

**Published:** 2026-04-14

**Authors:** Masato Kanazawa, Masahiro Hatakeyama, Toru Imamura, Tsutomu Kobayashi

**Affiliations:** 1 Department of Neurology, Niigata Neurosurgical Hospital, Niigata, Japan; 2 Faculty of Rehabilitation, Niigata University of Health and Welfare, Niigata, Japan; 3 Department of Neurology, Brain Research Institute, Niigata University, Niigata, Japan; 4 Department of Functional Neurology and Neurosurgery, Brain Research Institute, Niigata University, Niigata, Japan; 5 Department of Neurology, Niigata Rehabilitation Hospital, Niigata, Japan; 6 Department of Neurosurgery, Niigata Neurosurgical Hospital, Niigata, Japan

**Keywords:** Alzheimer’s disease, chronic rhinosinusitis, dementia clinics, dementia, posteriorcingulate cortex, SPECT

## Abstract

**Introduction:**

Single-photon emission computed tomography (SPECT) is widely used in dementia clinics to evaluate regional cerebral blood flow (rCBF). Posterior cingulate cortex (PCC) hypoperfusion is a supportive, though not definitive, marker for Alzheimer’s disease (AD). Magnetic resonance imaging (MRI)-defined sinus inflammation has been associated with systemic inflammation and altered brain connectivity; therefore, we aimed to determine whether MRI-defined chronic rhinosinusitis (CRS) is associated with differences in PCC perfusion patterns on SPECT among patients with cognitive impairment.

**Methods:**

We retrospectively reviewed 54 patients with cognitive impairment who had undergone brain MRI and SPECT. CRS was defined using MRI-based modified Lund–Mackay scores. SPECT findings were analyzed using the easy Z-score Imaging System (eZIS), focusing on PCC severity, extent, and ratio. Comparisons were performed between patients with and without CRS.

**Results:**

Ten patients (18.5%) had CRS. The frequency of AD was higher in patients with CRS than in patients without CRS (*p* = 0.028). Compared with patients without CRS (n = 44), those with CRS showed significantly greater PCC hypoperfusion: eZIS severity (1.7 ± 0.5 vs. 1.2 ± 0.4, *p* = 0.026), extent (26.1 ± 13.4% vs. 15.1 ± 14.3%, *p* = 0.196), and ratio (5.0 ± 2.8 vs. 2.0 ± 1.7, *p* = 0.013). No differences were observed in the cingulate island sign score (CIScore; *p* = 0.215). Moreover, in the subgroup of patients clinically diagnosed with AD, those with CRS showed significantly greater PCC hypoperfusion than those without CRS (1.8 ± 0.3 vs. 1.4 ± 0.5; *p* = 0.023). PCC hypoperfusion in CRS overlapped with canonical AD patterns but was not observed in non-AD dementias.

**Conclusion:**

Our exploratory findings suggest that MRI-defined CRS may be associated with differences in SPECT-derived PCC perfusion patterns in patients with cognitive impairment. Awareness of CRS as a common incidental MRI finding may help neurologists interpret SPECT results more cautiously in memory clinic settings.

## 1 Introduction

Neurodegenerative dementias, particularly Alzheimer’s disease (AD), are characterized by specific patterns of cerebral hypoperfusion, which can be detected by single-photon emission computed tomography (SPECT) (1, 2). A well-established finding in AD is hypoperfusion in the posterior cingulate cortex (PCC), a key hub of the default mode network (DMN) (3). Despite its diagnostic relevance, brain perfusion SPECT alone does not reliably distinguish among dementia subtypes, particularly in early or atypical presentations (4, 5). A significant proportion of patients with clinically suspected AD do not exhibit PCC abnormalities on SPECT. Therefore, SPECT is currently regarded as a supplementary tool that provides supportive—rather than definitive—evidence of neurodegeneration. According to the National Institute on Aging–Alzheimer’s Association (NIA-AA) 2024 research framework, SPECT is excluded from the core biomarkers of AD pathology (such as amyloid and tau) (6). SPECT is thus regarded as a non-core, adjunctive tool for clinical assessment; however, it is not used to biologically define AD.

Chronic rhinosinusitis (CRS) is a common inflammatory condition defined by the persistent inflammation of the nasal and paranasal mucosa lasting more than 12 weeks. Although traditionally viewed as a localized ear, nose, and throat (ENT) disorder, recent evidence suggests that CRS may have systemic effects, particularly through chronic low-grade inflammation and immune activation (7). The meningeal lymphatic and glymphatic systems, which drain waste molecules such as amyloid and tau, may also provide an association between ENT pathology and homeostasis. This is because the meningeal lymphatic vessels and glymphatic system are located near the ENT region, specifically within the meninges and around blood vessels in mice (8). These systems facilitate cerebrospinal fluid (CSF) and interstitial fluid movement to clear metabolic waste from the brain. Reportedly, CRS is associated with subjective cognitive impairment, slower reaction times, and accelerated cognitive decline, as measured by the Mini-Mental State Examination (MMSE), even in patients with dementia or mild cognitive impairment (MCI) (9). Furthermore, functional MRI studies have consistently demonstrated reduced connectivity and activity in the DMN, including the PCC and precuneus, in patients with CRS (10, 11). However, no studies have yet examined whether CRS modifies perfusion patterns on SPECT.

These findings suggest that CRS may modify cerebral perfusion signals in regions commonly evaluated in dementia imaging, such as the PCC, potentially influencing the interpretation of brain perfusion SPECT findings. While CRS itself is not a recognized risk factor for dementia subtypes, such as dementia with Lewy bodies (DLB) or frontotemporal dementia (FTD), its systemic inflammatory burden may modify neurovascular function or glymphatic clearance pathways, particularly in aging or cognitively vulnerable individuals.

This study retrospectively assessed whether CRS increases the likelihood or severity of PCC hypoperfusion on SPECT in patients with cognitive impairment or suspected dementia, particularly in early-stage or atypical cases, with the aim of determining whether CRS modifies SPECT findings in patients with cognitive impairment.

## 2 Methods

### 2.1 Standard protocol approvals, registration, and patient consent

This retrospective study was approved by the Ethics Committee of the Niigata Neurosurgical Hospital. Informed consent was waived due to the study’s retrospective design.

### 2.2 Study design

A retrospective analysis was conducted using the medical records of consecutive patients who underwent SPECT at Niigata Neurosurgical Hospital between March 2018 and July 2025. All patients were evaluated by neurologists trained and certified by the Japanese Society of Neurology and/or the American Academy of Neurology.

### 2.3 Study population

Patients with cognitive impairment who were referred to our outpatient clinic for SPECT evaluation were included. Clinical information and neuroradiological results were extracted from their medical records. Specifically, we collected data on age, sex, medical history, and disease duration. Cognitive function was assessed using the MMSE, the Japanese version of the Alzheimer’s Disease Assessment Scale-cognitive component (ADAS-Jcog), and the Clinical Dementia Rating (CDR) scale, where available. Patients with MCI and AD were diagnosed using the Diagnostic and Statistical Manual of Mental Disorders, Fifth Edition (DSM-5) criteria (12). DLB, behavioral variant FTD (bvFTD), progressive supranuclear palsy (PSP), and corticobasal syndrome (CBS) were diagnosed according to the established criteria (13–16). Patients classified as “clinically probable” or with a non-specific diagnosis were also included. The exclusion criteria were the presence of acute rhinosinusitis, a history of cranial trauma, or stroke within the preceding 6 months.

### 2.4 SPECT imaging analysis using the easy Z-score imaging system (eZIS) program

Brain perfusion SPECT was performed using 99mTc-ethyl cysteinate dimer (ECD). After intravenous injection of 99mTc-ECD (600 MBq, PDR Pharma Co., Ltd., Kyobashi, Tokyo, Japan), its passage from the heart to the brain was monitored using a rectangular gamma camera (Discovery NM 630, GE Healthcare, Hino, Tokyo). The Patlak plot method was used on 99mTc-ECD cerebral blood perfusion SPECT to measure mean global cerebral blood flow (CBF). The SPECT images were anatomically standardized using an original 99mTc-ECD template within the eZIS program. A Z-score map for each SPECT image was generated by comparing it with the mean and standard deviation (SD) of age-matched normal controls incorporated into the eZIS program as a normal control database. A voxel-by-voxel Z-score analysis was performed after voxel normalization to global means or cerebellar values; Z-score = ([control mean] − [individual value]) / (control SD). In the eZIS program, a specific region showing decreased regional CBF (rCBF) in very early AD, determined by Statistical Parametric Mapping analysis, was incorporated into an automated analysis of Z-score values as a volume of interest (VOI). A specific VOI was determined by a group comparison of 99mTc-ECD SPECT images between patients with very early AD and age-matched healthy volunteers. This VOI can be set in the PCC, precuneus, or parietal association cortex in the eZIS program. Three indicators used to discriminate between patients with very early AD and healthy controls were automatically calculated according to previous reports (1, 17). Severity of significantly decreased rCBF in a specific brain region in very early AD patients was calculated as the average positive Z-score within the VOI. Extent was defined as the percentage of coordinates with the Z-score exceeding the threshold value of 2 in the VOI. Ratio was calculated as the extent of a region showing a significant rCBF reduction in the VOI to the extent of a region showing a significant rCBF reduction in the whole brain was calculated, that is, the percentage of coordinates with a Z-value exceeding the threshold value of 2. This ratio indicates the specificity of rCBF reduction in the VOI compared with that of the whole brain. The cutoff values for discrimination between groups were 1.19, 14.2%, and 2.22 for severity, extent, and ratio, respectively (17). The cingulate island sign score (CIScore) was calculated by dividing the sum of the Z-scores of the hypoperfused region centered on the PCC, excluding the specific volume of interest analysis, by the sum of the Z-scores of the hypoperfused region in the specific volume of interest analysis of DLB patients (threshold value of 0.281) (18).

### 2.5 MRI-defined CRS

**Figure 1:**
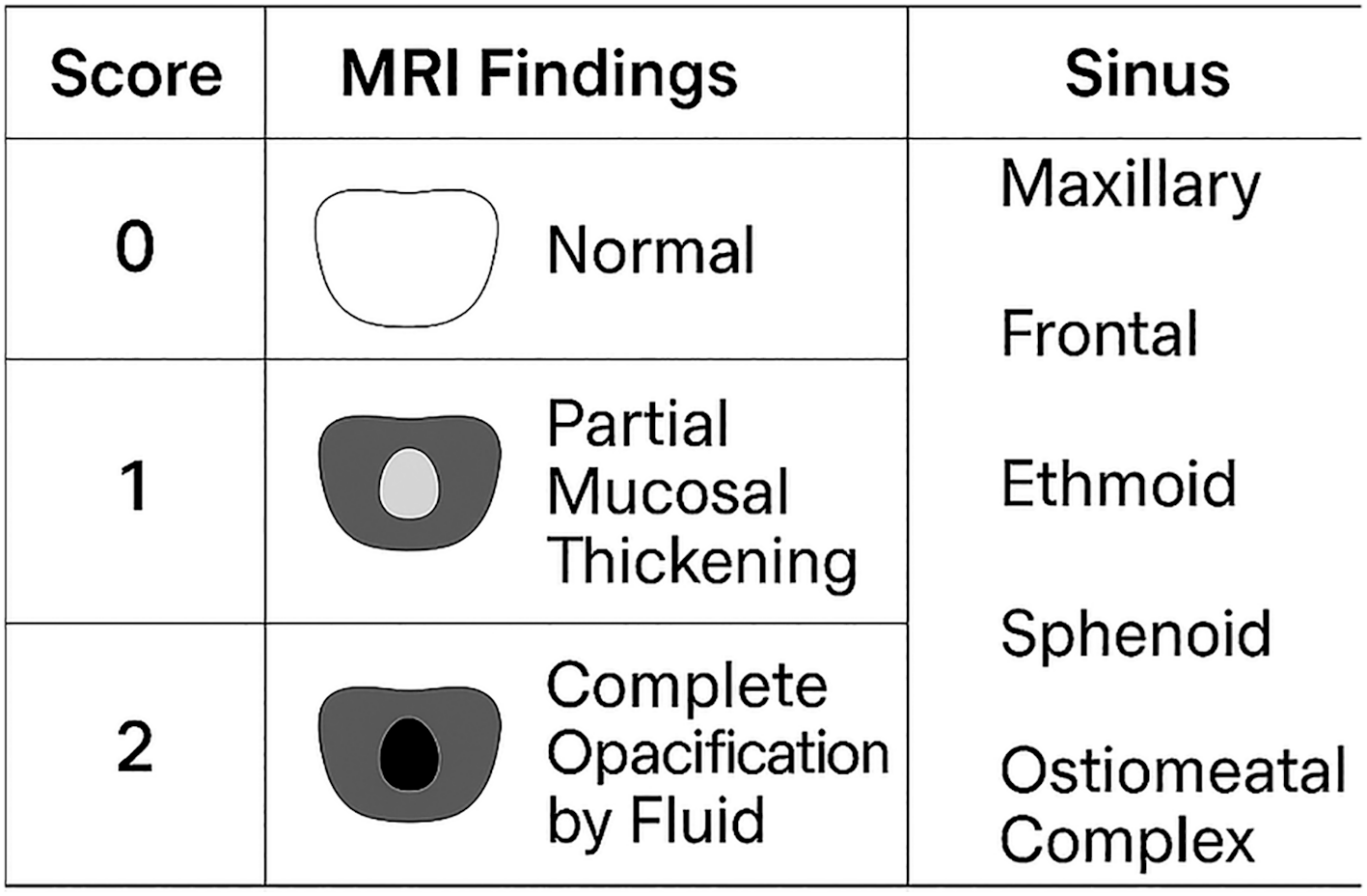
Modified Lund–Mackay score (mLMS) system using MRI. The mLMS scores of each bilateral sinus (maxillary, frontal, ethmoid, and sphenoid) (0, none; 1, partial; 2, complete) and the osteomeatal complex (0, none; 1, complete) based on the degree of opacification.

CRS classification in this study was based solely on MRI findings using the modified Lund–Mackay Score (mLMS). Patients were classified as MRI-defined CRS–positive (mLMS ≥ 4) or MRI-defined CRS–negative (9, 19). The mLMS assigns scores for each of the bilateral sinuses (maxillary, frontal, ethmoid, and sphenoid; 0 = none, 1 = partial, 2 = complete) and the osteomeatal complex (0 = none, 1 = complete) based on the degree of sinus opacification (Figure 1). MRI was performed using a 1.5- or 3-Tesla superconductive magnet. T2-weighted images were used to evaluate sinus mucosal thickening and opacification.

Notably, this definition reflects radiological sinus opacification and does not include systematic symptom assessment, endoscopic evaluation, or otolaryngological confirmation. As such, the term “MRI-defined CRS” in the present study refers to imaging-based sinus inflammation rather than clinically confirmed CRS.

### 2.6 Statistical analysis

Quantitative data were expressed as either the mean or mean ±SD [95% confidence interval]. Categorical variables were compared using *χ*^2^ tests. Continuous variables were compared using Student’s t-test or Welch’s t-test, depending on variance equality. Effect sizes were calculated using Cohen’s d test. The normality of continuous variables was assessed using the Shapiro–Wilk test, while the homogeneity of variance was evaluated using Levene’s test, prior to parametric comparisons. When normality assumptions were not met, non-parametric Mann–Whitney U-tests were applied. All statistical analyses were performed using IBM SPSS Statistics for Windows, version 25.0 (Armonk, NY, USA), and a *p*-value of < 0.05 was considered statistically significant.

## 3 Results

### 3.1 Patients’ characteristics

Clinical data are shown in Table 1. We reviewed the medical records of 54 consecutive Japanese patients referred to a dementia clinic. In this cohort, 10 (18.5%) patients had CRS. The differences in disease duration, MMSE score, ADAS-Jcog score, and CDR between patients with CRS and patients without CRS were not significant (Table 1). Although most patients with CRS were diagnosed with AD, those without CRS were diagnosed with AD or other dementia-related diseases. The frequency of AD was higher in patients with CRS than in patients without CRS (*p* = 0.028).

**Table 1:** Patients’ characteristics.

	CRS (+)	CRS (−)	95% confidence interval	*p*-value
Number	10	44		
Age, years	73.8 ± 6.0	75.3 ± 8.0	−6.3 to 3.3	0.516
Men (%)	10 (100%)	34 (77.2%)		0.174
Duration, years	2.5 ± 1.7	3.0 ± 1.6	−1.8 to 0.8	0.398
MMSE	22.3 ± 3.1	23.0 ± 4.9	−3.3 to 1.9	0.591
ADAS-Jcog	8.9 ± 3.4	9.7 ± 4.9	−4.0 to 2.4	0.591
CDR	0.8 ± 0.3	0.8 ± 0.4	−0.2 to 0.3	0.935
Diagnosis				0.028
AD	9 (90.0%)	23 (52.3%)		
Non-AD	1 (10.0%)	21 (47.7%)		

CRS, chronic rhinosinusitis; MMSE, Mini-Mental State Examination; AD, Alzheimer’s disease; ADAS-Jcog, Assessment Scale-cognitive component-Japanese version; CDR, clinical dementia rating.

The non-AD diagnoses in the CRS-negative group included two patients with mild cognitive impairment, five with frontotemporal dementia, four with progressive supranuclear palsy or corticobasal syndrome, two with dementia with Lewy bodies, one with vascular dementia, and seven undetermined diagnoses.

### 3.2 SPECT findings

**Figure 2:**
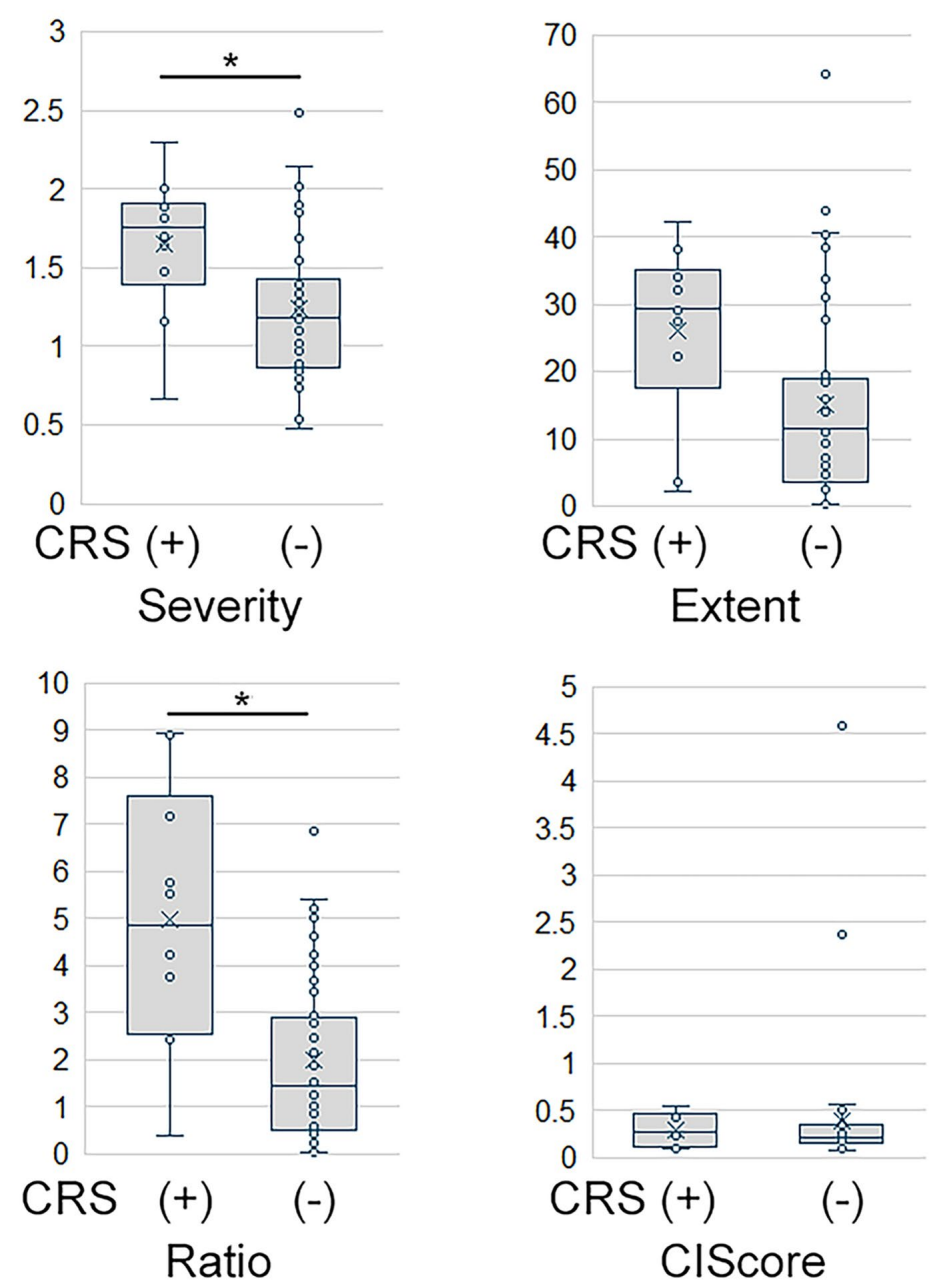
Box plots of SPECT findings. CIScore, cingulate island sign score; CRS, chronic rhinosinusitis.

The eZIS severity score did not significantly deviate from normality (*p* = 0.158); however, the extent, ratio, and CIScore all showed non-normal distributions (*p* < 0.001 for all). Accordingly, Welch’s t-test was applied for severity, while the Mann–Whitney U-tests were used to assess extent, ratio, and CIScore. Patients with CRS showed significantly greater PCC hypoperfusion than those without CRS, as reflected by higher severity (*p* = 0.026, Cohen’s d = 1.05) and ratio (*p* = 0.013). The difference in extent did not reach statistical significance (*p* = 0.196). The CIScore did not differ between groups (*p* = 0.215). (Table 2, Figure 2).

**Table 2:** Single-photon emission computed tomography findings.

	CRS (+)	CRS (−)	95% confidence interval	*p*-value
Severity	1.7 ± 0.5	1.2 ± 0.4	0.6 to 0.8	0.026
Extent	26.1 ± 13.4	15.1 ± 14.3	0.8 to 21.2	0.196
Ratio	5.0 ± 2.8	2.0 ± 1.7	1.6 to 4.4	0.013
CIScore	0.3 ± 0.2	0.4 ± 0.7	−0.3 to 0.2	0.215

CIScore, cingulate island sign score; CRS, chronic rhinosinusitis.

Moreover, in the subgroup of patients clinically diagnosed with AD, those with CRS showed significantly greater PCC hypoperfusion than those without CRS (1.8 ± 0.3 vs. 1.4 ± 0.5; *p* = 0.023, Welch’s t-test), and the effect size remained large (Cohen’s d = 0.99).

## 4 Discussion

Our study demonstrated that SPECT detected PCC hypoperfusion in cognitively impaired patients with CRS. Using quantitative eZIS analysis, CRS-positive patients showed significantly higher severity and ratio values within PCC volumes of interest compared with CRS-negative patients, despite comparable clinical cognitive test scores. This finding raises the possibility that MRI-defined CRS may be associated with differences in perfusion patterns among patients with dementia, with potential implications for diagnostic interpretation in memory clinics.

SPECT remains widely available and cost-effective for assessing rCBF, particularly in clinical settings where PET or CSF biomarkers are inaccessible (6) In AD, characteristic hypoperfusion in the PCC, precuneus, and temporoparietal cortices aligns with early cognitive deficits. However, the sensitivity of SPECT is limited: not all patients with AD show PCC involvement, and similar perfusion changes may occur in conditions such as depression, sleep disorders, or vascular disease. This variability underscores SPECT’s supportive yet non-definitive role in dementia diagnosis, consistent with its exclusion from core Alzheimer’s biomarkers in the 2024 NIA-AA research framework (6)

Nonetheless, SPECT contributes meaningfully to differential diagnosis. The “cingulate island sign” (relative PCC preservation with occipital hypoperfusion) supports DLB, Imabayashi et al. (18) while FTD shows frontal and anterior temporal hypoperfusion, sparing the PCC. Vascular dementia demonstrates patchy or asymmetric deficits. Thus, although not pathognomonic, SPECT remains informative when interpreted in clinical contexts.

CRS is traditionally viewed as a localized sinonasal disorder; however, increasing evidence links CRS to systemic inflammation, endothelial dysfunction, and altered neuroimmune signaling. MRI-defined sinus opacification, which is detectable as mucosal thickening or obstruction on MRI, is particularly common in older adults and may reflect persistent low-grade inflammation (20, 21). Functional MRI studies have shown CRS-related disruption of the DMN, particularly within the PCC and the precuneus. These regions overlap with SPECT’s diagnostic targets in AD, suggesting a plausible pathway by which CRS might influence brain perfusion.

Furthermore, the glymphatic and meningeal lymphatic systems, which bridge the ENT region and the central nervous system, provide additional mechanistic links. One is the cribriform pathway, in which cerebrospinal fluid and immune mediators drain along olfactory nerve bundles through the cribriform plate into nasal mucosal lymphatics, providing a direct interface with the ENT region (22). The other is the ventral skull base pathway, in which meningeal lymphatic vessels located along the basal dura convey cerebrospinal and interstitial fluid toward deep cervical lymph nodes (8, 23). While the former may be directly affected by sinonasal inflammation, the latter likely reflects a more global and chronic modulation of CNS fluid clearance. Inflammatory mediators associated with sinonasal inflammation may reach the brain through these channels and potentially influence neurovascular regulation or perfusion patterns. Accordingly, PCC hypoperfusion detected on SPECT may not always reflect primary neurodegeneration. In some patients, it may be influenced by CRS-related neuroinflammatory or glymphatic mechanisms, Yoon et al. (2) although this hypothesis remains speculative.

Our findings should also be considered in the context of large-scale epidemiological studies, which have generally not demonstrated an increased risk of dementia in CRS patients. A retrospective cohort of more than 10,000 patients reported no increased incidence of AD or other dementias in those with CRS compared with matched controls (24). Similarly, a Japanese MRI-based case–control study found no higher prevalence of sinus abnormalities in dementia patients than in control patients (25). However, these studies did not focus specifically on cognitively impaired patients undergoing SPECT evaluation. Therefore, our findings should be interpreted differently: CRS may not cause dementia; however, it may act as a modifier of imaging signals.

For neurologists, incidental CRS findings on MRI should not be ignored when interpreting SPECT. When PCC hypoperfusion is present in a patient with cognitive symptoms and CRS, clinicians should consider whether the finding reflects AD pathology, CRS-related inflammation, or both. Conversely, when perfusion deficits match the canonical bilateral temporoparietal and PCC pattern and correlate with memory impairment, AD remains the more likely explanation.

Integrating CRS assessment into dementia evaluations may therefore improve diagnostic precision. MRI-based modified Lund–Mackay scoring provides a simple way to quantify sinonasal inflammation. If the CRS burden is high, SPECT findings should be interpreted with caution, and ENT consultation may be warranted. Importantly, this approach highlights a potentially modifiable factor in dementia assessment: whether CRS treatment influences the inflammatory burden or cerebral perfusion currently remains unknown.

Quantitative SPECT tools, such as eZIS and three-dimensional stereotactic surface projection, improve objectivity by comparing patient scans to normative databases. These techniques reduce interobserver variability and allow for a more reliable detection of PCC and temporoparietal hypoperfusion (1, 26). Among the three eZIS indicators—severity, extent, and ratio—severity is generally considered to reflect pathological progression most sensitively, especially in the context of AD and MCI. The eZIS ratio metric reflects the relative burden of hypoperfusion within disease-specific regions compared with that of the entire brain, thereby partially mitigating the influence of global perfusion reductions (27). Nevertheless, absolute global CBF was not directly quantified in the present study. Accordingly, our findings are consistent with the possibility that MRI-defined CRS may be associated with altered SPECT perfusion patterns in regions commonly affected by AD, without implying a direct association with AD pathology itself. Although SPECT is still less precise than PET, its widespread availability makes it valuable in community- and resource-limited settings. In this context, considering CRS as a potential confounder becomes even more important because many centers may not have access to confirmatory amyloid or tau biomarkers.

Our study has several important limitations that warrant careful consideration. First, this was a retrospective study with a small overall sample size, and only 10 patients were classified as having CRS compared with 44 patients without CRS. This substantial group imbalance limits statistical power, increases the risk of type I and type II errors, and weakens the strength of our conclusions. Accordingly, the present findings should be interpreted as exploratory and hypothesis-generating rather than confirmatory. Second, CRS was defined exclusively based on MRI findings without systematic symptom assessment or ENT confirmation. Therefore, the present findings reflect MRI-defined sinus inflammation and cannot be directly generalized to clinically diagnosed CRS. Given the small number of CRS-positive patients, we did not perform multivariable regression analyses, as such adjustments would be statistically unstable and potentially misleading. Third, autopsy data were not available to confirm the underlying pathology, and amyloid or tau biomarkers were not used to establish AD diagnosis (6, 28, 29). In addition, multiple SPECT-derived metrics were analyzed without formal adjusting for multiple comparisons, which may increase the risk of type I errors. These factors constrain causal inferences and underscore the preliminary nature of the findings.

In conclusion, our exploratory findings suggest that MRI-defined CRS may be associated with differences in SPECT-derived perfusion patterns in the PCC in patients with cognitive impairment. Rather than indicating a direct etiological role, CRS may function as a clinical modifier or a potential confounder in functional neuroimaging-based dementia assessments. Awareness of CRS as a common incidental MRI finding may help neurologists interpret SPECT results more cautiously in memory clinics. Larger, prospective, biomarker-supported studies are required to validate these observations and determine their clinical relevance.

## Data Availability

The raw data supporting the conclusions of this article will be made available by the authors, without undue reservation.

## References

[1] Matsuda H Mizumura S Nagao T Ota T Iizuka T Nemoto K . Automated discrimination between very early Alzheimer disease and controls using an easy Z-score imaging system for multicenter brain perfusion single-photon emission tomography. AJNR Am J Neuroradiol. (2007) 28:731–6. 17416830 PMC7977345

[2] Yoon HJ Park KW Jeong YJ Kang D-Y. Significant correlation between cerebral hypoperfusion and neuropsychological assessment scores of patients with mild cognitive impairment. Nucl Med Commun. (2012) 33:848–58. doi: 10.1097/MNM.0b013e32835587f8, 22692580

[3] Leech R Sharp DJ. The role of the posterior cingulate cortex in cognition and disease. Brain. (2014) 137:12–32. doi: 10.1093/brain/awt162, 23869106 PMC3891440

[4] Dougall NJ Bruggink S Ebmeier KP. Systematic review of the diagnostic accuracy of 99mTc-HMPAO-SPECT in dementia. Am J Geriatr Psychiatry. (2004) 12:554–70. doi: 10.1176/appi.ajgp.12.6.554, 15545324

[5] Ferrando R Damian A. Brain SPECT as a biomarker of neurodegeneration in dementia in the era of molecular imaging: still a valid option? Front Neurol. (2021) 12:629442. doi: 10.3389/fneur.2021.629442, 34040574 PMC8141564

[6] Jack CR Jr Andrews JS Beach TG Buracchio T Dunn B Graf A . Revised criteria for diagnosis and staging of Alzheimer's disease: Alzheimer's Association workgroup. Alzheimers Dement. (2024) 20:5143–69. doi: 10.1002/alz.13859, 38934362 PMC11350039

[7] Bachert C Pawankar R Zhang L Bunnag C Fokkens WJ Hamilos DL . ICON: chronic rhinosinusitis. World Allergy Organ J. (2014) 7:25. doi: 10.1186/1939-4551-7-25, 25379119 PMC4213581

[8] Jin H Yoon JH Hong SP Hwang YS Yang MJ Choi J . Increased CSF drainage by non-invasive manipulation of cervical lymphatics. Nature. (2025) 643. doi: 10.1038/s41586-025-09052-5, 40468071 PMC12267054

[9] Jung HJ Lee JY Choi YS Choi H-G Wee J-H. Chronic rhinosinusitis and progression of cognitive impairment in dementia. Eur Ann Otorhinolaryngol Head Neck Dis. (2021) 138:147–51. doi: 10.1016/j.anorl.2020.05.017, 33041243

[10] Jafari A de Lima Xavier L Bernstein JD Simonyan K Bleier BS. Association of sinonasal inflammation with functional brain connectivity. JAMA Otolaryngol Head Neck Surg. (2021) 147:534–43. doi: 10.1001/jamaoto.2021.0204, 33830194 PMC8033506

[11] Zhang Z Wu Y Luo Q Tu J Li J Xiong J . Regional homogeneity alterations of resting-state functional magnetic resonance imaging of chronic rhinosinusitis with olfactory dysfunction. Front Neurosci. (2023) 17:1146259. doi: 10.3389/fnins.2023.1146259, 37575305 PMC10412925

[12] American Psychiatric Association. Diagnostic and Statistical Manual of Mental Disorders. 5th ed. Arlington, VA: American Psychiatric Association (2013).

[13] McKeith IG Boeve BF Dickson DW Halliday G Taylor J-P Weintraub D . Diagnosis and management of dementia with Lewy bodies: fourth consensus report of the DLB consortium. Neurology. (2017) 89:88–100. doi: 10.1212/WNL.0000000000004058, 28592453 PMC5496518

[14] Rascovsky K Hodges JR Knopman D Mendez MF Kramer JH Neuhaus J . Sensitivity of revised diagnostic criteria for the behavioural variant of frontotemporal dementia. Brain. (2011) 134:2456–77. doi: 10.1093/brain/awr179, 21810890 PMC3170532

[15] Höglinger GU Respondek G Stamelou M Kurz C Josephs KA Lang AE . Clinical diagnosis of progressive supranuclear palsy: the movement disorder society criteria. Mov Disord. (2017) 32:853–64. doi: 10.1002/mds.26987, 28467028 PMC5516529

[16] Armstrong MJ Litvan I Lang AE Bak TH Bhatia KP Borroni B . Criteria for the diagnosis of corticobasal degeneration. Neurology. (2013) 80:496–503. doi: 10.1212/WNL.0b013e31827f0fd1, 23359374 PMC3590050

[17] Takemaru M Kimura N Abe Y Goto M Matsubara E. The evaluation of brain perfusion SPECT using an easy Z-score imaging system in the mild cognitive impairment subjects with brain amyloid-β deposition. Clin Neurol Neurosurg. (2017) 160:111–5. doi: 10.1016/j.clineuro.2017.06.018, 28715708

[18] Imabayashi E Soma T Sone D Tsukamoto T Kimura Y Sato N . Validation of the cingulate island sign with optimized ratios for discriminating dementia with Lewy bodies from Alzheimer's disease using brain perfusion SPECT. Ann Nucl Med. (2017) 31:536–43. doi: 10.1007/s12149-017-1181-4, 28547521 PMC5517560

[19] Lin HW Bhattacharyya N. Diagnostic and staging accuracy of magnetic resonance imaging for the assessment of sinonasal disease. Am J Rhinol Allergy. (2009) 23:36–9. doi: 10.2500/ajra.2009.23.3260, 19379610

[20] Shi JB Fu QL Zhang H Cheng L Wang YJ Zhu DD . Epidemiology of chronic rhinosinusitis: results from a cross-sectional survey in seven Chinese cities. Allergy. (2015) 70:533–9. doi: 10.1111/all.12577, 25631304 PMC4409092

[21] Orb Q Pulsipher A Smith KA Ashby S Alt JA. Correlation between systemic inflammatory response and quality of life in patients with chronic rhinosinusitis. Int Forum Allergy Rhinol. (2019) 9:458–65. doi: 10.1002/alr.22289, 30657646 PMC6491244

[22] Louveau A Smirnov I Keyes TJ Eccles JD Rouhani SJ Peske JD . Structural and functional features of central nervous system lymphatic vessels. Nature. (2015) 523:337–41. doi: 10.1038/nature14432, 26030524 PMC4506234

[23] Ahn JH Cho H Kim JH Kim SH Ham J-S Park I . Meningeal lymphatic vessels at the skull base drain cerebrospinal fluid. Nature. (2019) 572:62–6. doi: 10.1038/s41586-019-1419-5 31341278

[24] Son DS Kim JI Kim DK. A longitudinal study investigating whether chronic rhinosinusitis influences the subsequent risk of developing dementia. J Pers Med. (2024) 14:1081. doi: 10.3390/jpm14111081, 39590573 PMC11595754

[25] Yasue M Sugiura S Uchida Y Otake H Teranishi M Sakurai T . Prevalence of sinusitis detected by magnetic resonance imaging in subjects with dementia or Alzheimer's disease. Curr Alzheimer Res. (2015) 12:1006–11. doi: 10.2174/1567205012666150710105152 26159202

[26] Ishii K Kanda T Uemura T Miyamoto N Yoshikawa T Shimada K . Computer-assisted diagnostic system for neurodegenerative dementia using brain SPECT and 3D-SSP. Eur J Nucl Med Mol Imaging. (2009) 36:831–40. doi: 10.1007/s00259-008-1051-3, 19148640

[27] Tokumitsu K Yasui-Furukori N Takeuchi J Yachimori K Sugawara N Terayama Y . The combination of MMSE with VSRAD and eZIS has greater accuracy for discriminating mild cognitive impairment from early Alzheimer's disease than MMSE alone. PLoS One. (2021) 16:e0247427. doi: 10.1371/journal.pone.0247427, 33617587 PMC7899318

[28] Montoliu-Gaya L Salvadó G Therriault J Nilsson J Janelidze S Weiner S . Plasma tau biomarkers for biological staging of Alzheimer's disease. Nat Aging. (2025) 5:2297–308. doi: 10.1038/s43587-025-00951-w, 40847200 PMC12618263

[29] Ossenkoppele R Jansen WJ Rabinovici GD Knol DL van der Flier WM van Berckel BNM . Prevalence of amyloid PET positivity in dementia syndromes: a meta-analysis. JAMA. (2015) 313:1939–49. doi: 10.1001/jama.2015.4669, 25988463 PMC4517678

